# Postnatal development of mouse spermatogonial stem cells as determined by immunophenotype, regenerative capacity, and long-term culture-initiating ability: a model for practical applications

**DOI:** 10.1038/s41598-024-52824-8

**Published:** 2024-01-27

**Authors:** Youngmin Song, Xiangfan Zhang, Joëlle A. Desmarais, Makoto Nagano

**Affiliations:** 1 Department of Obstetrics and Gynecology, McGill University, and the Child Health and Human Development Program, The Research Institute of the McGill University Health Centre, 1001 Decarie Boulevard, Rm# EM0.2212, Montreal, QC H4A 3J1 Canada; 2 Present Address: JEFO Nutrition Inc, 5020 Avenue Jefo, Saint-Hyachinthe, Quebec, J2R 2E7 Canada

**Keywords:** Cell biology, Developmental biology, Stem cells

## Abstract

Spermatogonial stem cells (SSCs) are the foundation of life-long spermatogenesis. While SSC research has advanced greatly over the past two decades, characterization of SSCs during postnatal development has not been well documented. Using the mouse as a model, in this study, we defined the immunophenotypic profiles of testis cells during the course of postnatal development using multi-parameter flow cytometry with up to five cell-surface antigens. We found that the profiles progress over time in a manner specific to developmental stages. We then isolated multiple cell fractions at different developmental stages using fluorescent-activated cell sorting (FACS) and identified specific cell populations with prominent capacities to regenerate spermatogenesis upon transplantation and to initiate long-term SSC culture. The data indicated that the cell fraction with the highest level of regeneration capacity exhibited the most prominent potential to initiate SSC culture, regardless of age. Interestingly, refinement of cell fractionation using GFRA1 and KIT did not lead to further enrichment of regenerative and culture-initiating stem cells, suggesting that when a high degree of SSC enrichment is achieved, standard markers of SSC self-renewal or commitment may lose their effectiveness to distinguish cells at the stem cell state from committed progenitors. This study provides a significant information resource for future studies and practical applications of mammalian SSCs.

## Introduction

Spermatogonial stem cells (SSCs) drive the high-throughput, life-long production of sperm. While these cells play critical roles to maintain the genetic integrity and the health of offspring across generations, SSCs also provide an important resource for male fertility preservation and restoration^1,2^. Gonadotoxic treatments, such as anti-cancer therapies, can induce permanent male infertility, and the only option to preserve male fertility is currently sperm cryopreservation^2^. However, prepubertal boys cannot benefit from this option, as they do not produce sperm at the time of therapy. Since SSCs are present from the time of birth, a scheme to harvest SSCs before gonadotoxic therapy, and following cryopreservation, transplant them back to a patient afterwards has been widely anticipated to become an effective strategy to safeguard the fertility of the boys whose reproductive ability is at risk before reaching puberty^2^.

Multiple technical challenges exist to make this SSC-based fertility preservation strategy a clinical reality. One is a possibility that transplantation of SSCs harvested before anti-cancer therapies re-introduces tumorigenic cells into cancer survivors^3–5^. This concern can be overcome by purifying SSCs or purging tumorigenic cells. We thus need to achieve a sufficiently high degree of SSC purification before transplantation. Another challenge is the rarity of SSCs in testes. As seen in many stem cell types, SSCs represent a small fraction of the cell lineage to which they belong^6^. In mice, it has been reported that 0.01 – 0.02% of adult cells in the seminiferous epithelium are SSCs, as functionally determined using spermatogonial transplantation^7,8^. This problem is further compounded by the small size of testicular biopsies derived from young boys, which will be the source of SSCs for fertility preservation. It is widely anticipated, therefore, that human SSCs will need to be propagated in vitro^9,10^. Such SSC cultures may also effectively lead to a demise of tumorigenic cells prior to transplantation through the environment preferable for SSC expansion.

Developing these techniques of human SSC isolation and culture is not a straightforward process, however. First, SSCs are detected by their regenerative function, but research to determine the functionality of SSCs using spermatogonial transplantation is not feasible in humans. It is thus difficult to monitor the results of human SSC isolation and enrichment. Second, culture techniques of human SSCs have been reported in the past^11–13^, but they have not been developed sufficiently for clinical applications. More fundamentally, the experimental use of testis tissues derived from prepubertal boys for technical development can be ethically controversial. Considering that the major beneficiaries of SSC-based male fertility preservation and restoration are young boys, these perspectives indicate the need to establish an animal model that provides important information about the characteristics and process of SSC development after birth.

SSCs are also expected to become an important resource for reproduction of livestock species^14–16^. The maintenance, production, and propagation of favored traits of livestock can be efficiently done based on the use of SSCs. Similar to human SSCs, however, studies and applications of livestock SSCs encounter various challenges, including the fact that spermatogonial transplantation is not a practical option to assay SSC functions^14–16^.

Notably, mouse SSCs regenerate spermatogenesis upon transplantation and can be propagated efficiently in vitro while they are reported to express some cell-surface antigens that are shared with undifferentiated spermatogonia of other mammalian species, including humans^17–19^. Using the mouse as a model, in this study, we extensively analyzed the developmental process of regenerative and culture-initiating stem cells based on multi-parameter flow cytometry, spermatogonial transplantation, and SSC culture. We then determined the progression of immunophenotypes (i.e., flow cytometric profiles) of regenerative and culture-initiating cells during the prepubertal period. Importantly, our data indicate that regenerative and culture-initiating stem cells are isolated into the same cell fraction at a significantly high purity compared to those reported in the past and thus suggest that a cell population that has regenerative capacity can be identified by an in-vitro assay in an animal species where spermatogonial transplantation is difficult or unethical. This study also reveals that once enriched at a high level, SSC functions cannot be predicted clearly by the standard markers of SSC self-renewal and commitment, such as GFRA1 and KIT. The information presented here represents a critical knowledge resource for developing clinical and practical applications of SSCs.

## Results

### Prepubertal testes have smaller SSC pools but are highly enriched for SSCs compared to adult testes

Using spermatogonial transplantation, we first determined the developmental kinetics of SSC quantity during the postnatal development of mouse testes. Previous studies reported an increase in SSC concentrations from neonatal (P0-2) through pup (P6) to adult intact and cryptorchid testes^20,21^. When we measured SSC numbers by transplanting donor testis cells with no cell fractionation at different ages after birth, we detected, on average, 6.5, 39.0, 15.4, and 2.6 colonies of donor-derived spermatogenesis per 10^5^ donor cells transplanted at P0-2, P6-8, P16-18, and adult age, respectively (Supplementary Table S1). The results well correspond to those of past studies. By considering the number of total donor testis cells harvested before transplantation, we calculated that regenerative SSCs present in an entire testis produced 31.9 colonies upon transplantation at P0-2. Thereafter, the quantity of these SSCs increased with age to 278, 390, and 737 per testis from P6-8 through P16-18 to adult, indicating that the size of SSC pools in an entire testis rapidly increases during postnatal development in mice. However, since the testis enlarges during this period due to the establishment of spermatogenesis (Supplementary Table S1), we found that 1 mg of tissue from a prepubertal testis carried a significantly greater SSC pool, compared to 1 mg of adult testis tissue. Thus, 49.8 colonies of donor-derived spermatogenesis were derived per mg testis tissue at P0-2, which increased to 168.3 colonies at P6-8, followed by a decline to 28.8 and 7.3 colonies/mg testis tissue at P16-18 and adult, respectively (Supplementary Table S1). These data demonstrate that more SSCs can be harvested from a unit size of testis fragment before puberty than adult, even though the total SSC number in an entire testis drastically increases through postnatal development.

### Testis cells show age-dependent flow cytometric profiles after birth

We next examined the immunophenotypic profiles of testis cells during testis development after birth. To this end, testis cells at different ages were first selected using flow cytometry for low side-scatter levels, singlets, and viable cells (Supplementary Fig. S1). Previous studies have shown that SSCs show low levels of side scatter in flow cytometry^22,23^. Resulting cells were further processed for negative selection using cell-surface antigens, MHC-I, CD45, and CD74. MHC-I is known to be absent on spermatogonia at least at the protein level^23,24^. CD45 is a pan-hematopoietic marker while CD74 is a cell-surface HLA class II histocompatibility antigen gamma chain^25,26^. Subsequently, we characterized the cells at various stages of postnatal testis development in mice, using two cell-surface antigens that have been well established as SSC markers: THY1 and ITGA6. Past studies reported that THY1 is a cell-surface antigen that allows for the best SSC enrichment when used as a single parameter^23^, and ITGA6 is also effective for SSC enrichment^22,27^.

**Figure 1:**
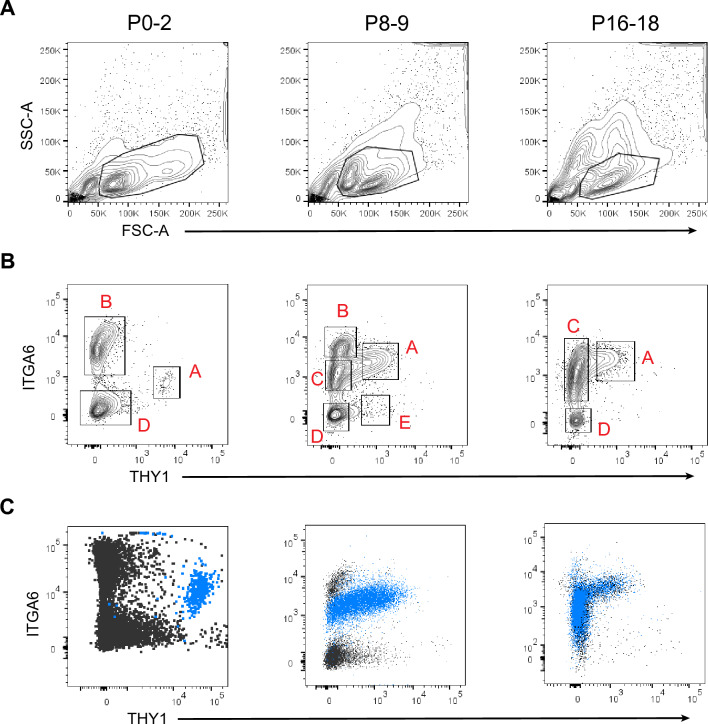
Flow cytometric profiles of mouse testis cells during postnatal development. (**A**) Profiles of forward scatter (FSC) and side scatter (SSC) of all events immediately after single cell preparation. Spermatogonial stem cells are known to localize to a cell fraction with low levels of side scatter (SSC)^22^. (**B**) Multiple cell fractions are identified by flow cytometry based on expression of THY1 and ITGA6. Progression of stage-specific profiles is evident during postnatal development. Fractions are labelled with black rectangles and red letters (Fractions A to E). To aid gating of Fractions A and C at P8-9, we used the “Fluorescence Minus One (FMO)” control for THY1 expression. We found that an ITGA6 FMO was unnecessary, as Fractions B and D were clearly separable manually^61^ and based on TRA98 staining (C). (**C**) Flow cytometric profiles of germ cells detected using intracellular flow cytometry with the TRA98 antibody. TRA98 + cells are colored blue. Note that germ cells in Fraction C are confined to the THY1-ITGA6-medium cell population at P8-9 but extend towards the THY1-ITGA6-high to -low profile at P16-18.

The flow cytometric profiling demonstrated dynamic shifts in immunophenotypes of testis cells throughout postnatal development (Fig. 1 and Supplementary Fig. S2). Overall, we noted up to five cell fractions, as seen at the P8-9 stage (Fig. 1), but the emergence and disappearance of individual fractions were stage-specific. For example, only three fractions were identified at P0-2, which we call Fractions A (THY1 + ITGA6 +), B (THY1– ITGA6^hi^), and D (THY1– ITGA6–). This three-fraction pattern persisted until P6, while the profile of Fraction A shifted gradually, with ITGA6 levels increasing and THY1 levels decreasing from P0 to P6 (Supplementary Fig. S2). The cell fraction that corresponds to C (THY1– ITGA6^mid^) becomes detectable by P8-9. Thus, all five fractions are observed at P8-9, including C (THY1– ITGA6^mid^) and E (THY1 + ITGA6–). From P8-9 to P16-18, Fraction C expands (14.9% of total cells plotted on the profile at P8-9 to 65.1% at P16-18), while Fraction B becomes undetectable by P16 (Fig. 1). The flow cytometric profiles are generally similar from P16 to adult (Supplementary Fig. S2). The adult profile shows a greater presence of THY1-cells while THY1 + ITGA6 + cells represent a smaller cell population. Based on these data, we determined that evident changes in the THY1/ITGA6 immunophenotype occurred between P4 and P6 as well as between P12 and P14 (Supplementary Fig. S2). We thus identified P0-2, P8-9, and P16-18 as three stages of postnatal testis development in mice with distinct, representative THY/ITGA6 profiles (Fig. 1), the stages which we characterized further.

It is possible that the cell fractions shown in Fig. 1 included multiple testis cell types. To identify germ cell fractions, we employed intracellular flow cytometry using the TRA98 antibody, which reacts with GENA110 (germ cell-specific nuclear antigen) that is expressed only in germ cells from the stages of primordial germ cells to round spermatids^28^. We found that the TRA98 signal at P0-2 was confined to Fraction A alone in which TRA98 + cells represented 94.8% of the cells (Fig. 1C). At P8-9, TRA98 + cells were found in Fractions A and C with 90.4% and 73.5% of each fraction, respectively. Germ cells were also restricted to Fractions A and C at P16-18, and 91.2% of Fraction A were TRA98 + cells while Fraction C, 89.6%. These results demonstrate that Fractions A and C are the germ cell fractions at all three stages of postnatal development.

### Regenerative stem cells are restricted to the THY1/ITGA6 double-positive cell fraction in prepubertal testes

**Figure 2:**
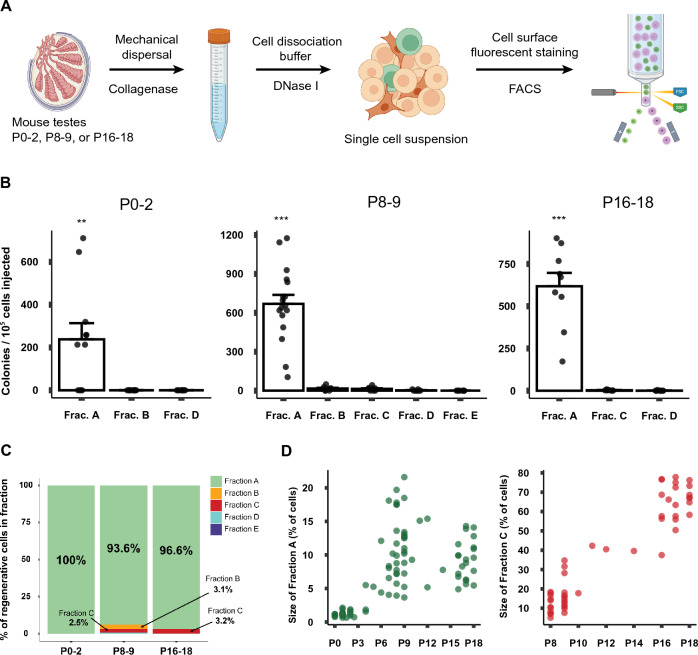
Fraction A contains nearly all regenerative stem cells regardless of age. (**A**) Schematic representation of the procedure from the preparation of a single cell suspension of testes to fractionation using FACS. (**B**) Regenerative capacity of each cell fraction at three developmental stages as determined using spermatogonial transplantation following FACS. Each point represents data from one recipient testis. The X-axis indicates each Fraction. Significant differences are denoted by asterisks (*p < 0.05; **p < 0.01; ***p < 0.001). (**C**) The proportion of SSCs detected in each cell fraction at different developmental stages. Data were calculated as SSC activity in each fraction (colony numbers/10^5^ cells transplanted) multiplied by the population size of each fraction (% of cells in each fraction relative to all cells presented on the THY1/ITGA6 flow profile). (**D**) Proportions of germ cells in Fraction A and Fraction C relative to an entire cell population examined at each stage of postnatal development. Note that the X-axis begins at different ages (P0 for Fraction A, P8 for Fraction C). Mean size of Fraction A at P0-2: 1.22%, P8-9: 11.51%, and P16-18: 9.27%. Mean size of Fraction C at P8-9 and P16-18: 14.86% and 65.12%. Linear regression lines of Fraction A from P0 to P18: y = 0.5 x + 3.3 (*p* < 0.001, R^2^ = 0.29); Fraction C from P8 to P18: y = 5.9x – 34.7 (*p* < 0.001, R^2^ = 0.89).

In order to identify the SSC fraction functionally, we sorted out each cell fraction across the three developmental stages using FACS and determined each fraction’s regenerative capacity by spermatogonial transplantation (Fig. 2A).

As clearly seen in Fig. 2B, Fraction A contained the vast majority of SSCs regardless of age. The magnitude of SSC frequency in Fraction A was similar at P8-9 and P16-18 (669.2 ± 69.0 and 618.1 ± 79.4 colonies per 10^5^ cells transplanted, respectively), while the frequency was lower at P0-2 (238.3 ± 75.4 colonies per 10^5^ cells transplanted). Compared to unsorted testis cells (Supplementary Table S1), Fraction A exhibited remarkably high levels of SSC enrichment at all stages (Supplementary Table S2). For instance, approximately 40-fold higher SSC enrichment was achieved at P0-2 and P16-18. Importantly, although a significant proportion of Fraction C represents germ cells at P8-9 and P16-18, only negligible levels of regenerative SSCs were detectable in this fraction (13.7 ± 4.2 colonies per 10^5^ cells transplanted at P8-9 and 2.9 ± 0.9 colonies per 10^5^ cells transplanted at P16-18, Fig. 2C). Since the cells in Fraction C do not express significant levels of THY1, the results suggest that the loss of THY1 expression apparently corresponds to the loss of the SSC state.

Notably, the levels of SSC enrichment achieved in this study are significantly greater than those reported in the past. As 12% of total SSCs transplanted are reported to actually regenerate spermatogenesis^7,29^, our data indicate that 1 in 50 cells in Fraction A is regenerative stem cells at P0-2, and this value increases to 1 SSC in 18 cells at P8-9 and P16-18 (Supplementary Table S2). This level of SSC enrichment at P8-9 is up to 3.7-fold higher than the levels reported in past studies using a genetic marker (eGFP)^30,31^ (Supplementary Table S3). At P0-2, it is roughly 14-fold higher than a previous study which used FACS^32^ and at P16-18 we found no comparable literature.

Figure 2D shows shifts in the proportion of Fractions A and C over time relative to all cells analyzed in the THY1/ITGA6 profiling. Fraction A increased in its population size from 1.2% at P0 to 11.5% at P8-9 and 9.3% at P16-18, while Fraction C expanded from 14.9% at P8-9 to 65.1% at P16-18. Linear regression analyses detected significant upward trends in both fractions (Fig. 2D), with more evident linearity seen with Fraction C (R^2^ = 0.29 for Fraction A vs. 0.89 for Fraction C). Together with the lack of regenerative capacity in Fraction C, these results demonstrate that non-regenerative cells expand in Fraction C during the establishment of first spermatogenesis after birth. We also speculate that regenerative cells in Fraction A may supply non-regenerative cells through their commitment to differentiation.

### Culture-initiating cells can be prospectively identified by flow cytometry

**Figure 3:**
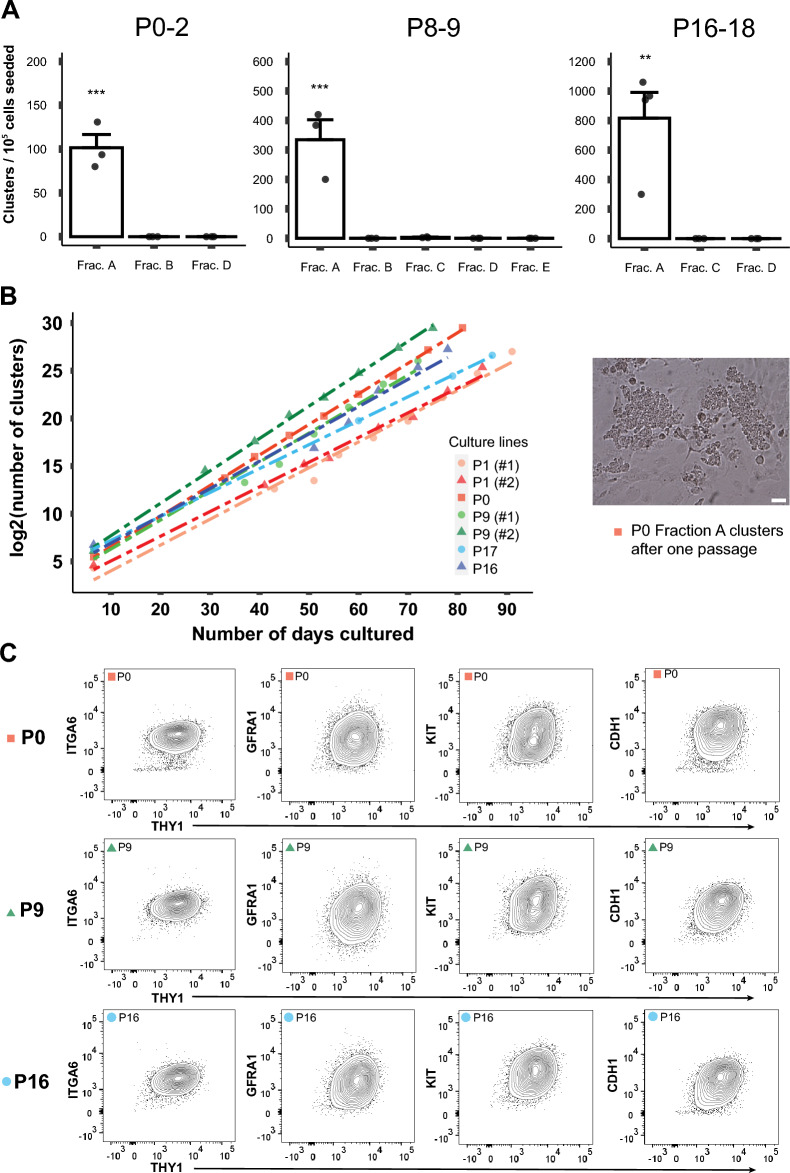
Cluster-initiating cells are predominantly represented by Fraction A throughout development after birth. (**A**) Number of clusters in each fraction detected using the CFA assay (short-term culture with no passaging). Nearly all cluster-initiating ability is localized to Fraction A. Statistical significances are indicated by asterisks (*p < 0.05; **p < 0.01; ***p < 0.001). (**B**) Long-term culture and expansion of seven cluster lines established using Fraction A cells at three different developmental stages. Cultures were continued for up to 91 days. The number of clusters after the first week was derived from Fig. 3A. Thereafter, the numbers were counted visually at each passage. (**C**) Representative flow cytometric profiles at each developmental stage. Culture lines were analyzed after 10 passages. Expression of five cell-surface markers were examined: THY1, ITGA6, GFRA1, KIT, and CDH1.

Although SSC culture is well established in mice, most studies employ spermatogonia derived from pups around 6 days of age to start the culture^33–36^. It is important to demonstrate that we are able to identify and select prospectively a specific cell population best suited for initiating SSC culture during postnatal development. To address this, we sorted each fraction of testis cells at the three stages of development (Fig. 1) and determined its ability to generate three-dimensional aggregates of spermatogonia (called “clusters”) in vitro, using the short-term, cluster-forming assay^36,37^ (Fig. 3). Data showed that similar to the regeneration capacity, the cluster-forming ability was detectable only in Fraction A at all the three stages of development. Although we detected cluster-forming activity in Fraction C at P8-9, its level was negligible (Fig. 3A). Fraction A at P16-18 showed the highest cluster-forming activity (816.7 clusters/10^5^ cells placed in culture), followed by P8-9 (334.5 clusters/10^5^ cells placed in culture) and P0-2 (101.5 clusters/10^5^ cells placed in culture). These data demonstrate that the cluster-forming activity increases during the establishment of the spermatogenesis after birth. Interestingly, even though the Fraction A cells showed near-identical levels of regenerative capacity upon transplantation at P8-9 and P16-18 (Fig. 2A), the cluster-forming ability of Fraction A in vitro at P16-18 was more than double of that at P8-9 (Fig. 3A). It is tempting to speculate that as postnatal development of spermatogenesis proceeds and more progenitors are generated and expanded, some of these progenitors might retain cluster-initiating ability after losing regenerative capacity.

Next, we established long-term culture lines from Fraction A cells at different ages. Clusters were maintained by continuously subculturing up to 11 passage generations for 72 to 91 days (Fig. 3B); characteristics of each culture line is shown in Supplementary Table S4. The results showed that even though cluster-forming ability in Fraction A differed across developmental stages (Fig. 3A), once established, clusters propagated at a similar rate regardless of the developmental stages (Fig. 3B). Thus, cluster doubling time was 3 to 4 days with all cluster lines established (Supplementary Table S4).

Given the similar doubling time of clusters regardless of age, we suspected that profiles of SSC marker expression in cluster cells may not be affected by age. We thus analyzed the expression of THY1, ITGA6, GFRA1, CDH1, and KIT in cluster cells established from Fraction A at different ages using flow cytometry. THY1, ITGA6, GFRA1, and CDH1 are considered as positive markers of mouse SSCs while KIT as a negative marker. The results showed that these five cell-surface proteins were similarly expressed on cluster cells regardless of age, and we were unable to detect distinguishable subfractions in the cultured cells. As such, clusters derived from P0-2 cells expressed KIT at the levels comparable to P8-9 and P16-18 clusters after long-term culture (Fig. 3C). This is interesting, since Fraction A cells, which are directly derived from testes, do not express KIT at P0-2 (i.e., before culture), in contrast to those cells at P8-9 and P16-18, which express KIT significantly in vivo (Supplementary Fig. S3, bottom). These results suggest that once established, cluster cells show similar levels of proliferation ability and patterns of SSC marker expression regardless of the developmental stages of origin.

### Expression of SSC marker genes is Fraction- and stage-specific during postnatal development

**Figure 4:**
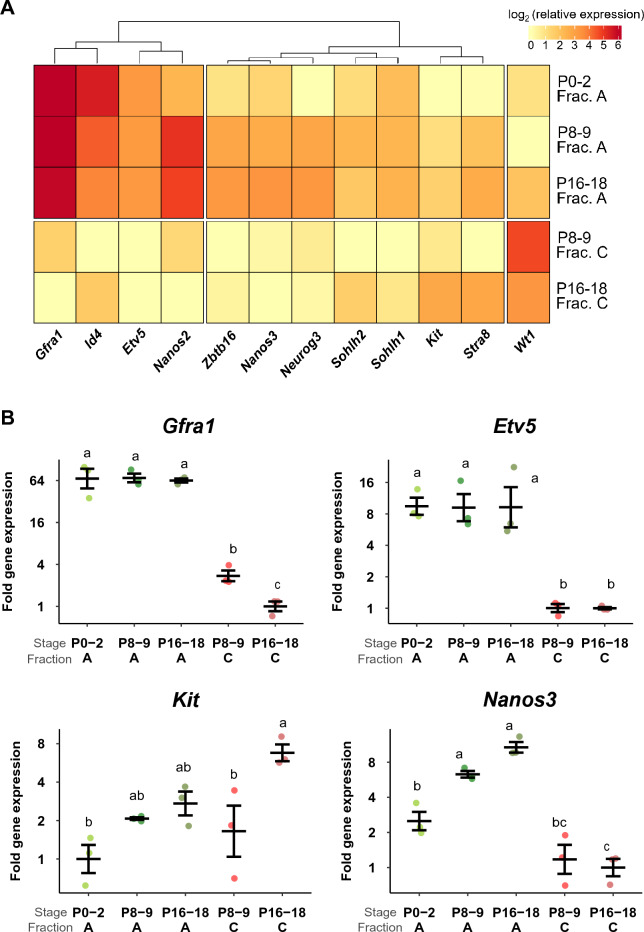
Expression of spermatogonial marker genes as determined using quantitative RT-PCR. (**A**) The expression level of each marker gene is shown in the heatmap format for Fractions A and C at three developmental stages. (**B**) Relative expression of four selected genes (*Gfra1*, *Etv5*, *Nanos3*, *Kit*) in Fractions A and C at the three developmental stages. Statistical differences are indicated by distinct alphabets (p < 0.05). For example, there was a significant difference detected between data labeled 'a' and 'bc', but there was no significant difference between data labeled 'bc' and 'c'.

We next determined expression profiles of spermatogonial genes in Fractions A and C in vivo, using quantitative PCR (Fig. 4 and Supplementary Fig. S4). We examined a panel of 12 genes, five of which are considered to be SSC/self-renewal markers (*Gfra1*, *Id4*, *Etv5*, *Nanos2*, and *Zbtb16*), four progenitor markers (*Nanos3*, *Neurog3*, *Sohlh1*, and *Sohlh2*), and two differentiation markers (*Kit* and *Stra8*). *Wt1*, a Sertoli cell marker^38^, was also included in the analysis. Figure 4A summarizes the results in a heatmap. Rows were grouped using the R package ‘pheatmap’, in order to identify sets of genes that had similar expression patterns^39^. Complete-linkage clustering was performed based on Euclidean distance. *Wt1* was excluded from the hierarchical clustering algorithm because it is uniquely a Sertoli cell marker.

The data showed that genes can be divided into three groups based on hierarchical clustering: Group 1 (*Gfra1*, *Id4*, *Etv5*, and *Nanos2*), Group 2 (*Zbtb16*, *Nanos3*, *Neurog3*, *Sohlh1*, *Sohlh2*), and Group 3 (*Kit*, *Stra8*). This grouping largely reflects the previous knowledge, with Group 1 as the SSC markers, Group 2 the progenitor markers, and Group 3 the differentiation markers. Although *Zbtb16* is generally considered to be a self-renewal marker^40,41^, it was grouped with progenitor markers in our analysis. This corresponds to the results of the study in which the Z*btb16* gene was classified as marker for progenitor cells^4,42^.

Most of the spermatogonia genes examined are more highly expressed in Fraction A than in Fraction C. *Kit* and *Stra8* were clearly detectable in Fraction C at P16-18 (Fig. 4A and B). These observations support the notion that cells in Fraction C are at an advanced stage of germ cell differentiation.

Compared to P0-2, Fraction A at P8-9 and P16-18 clearly showed greater expression levels of the progenitor and differentiation marker genes (Group 2, Fig. 4A), reflecting the ongoing progress of first spermatogenesis after birth. Focusing on Group 1 genes (self-renewal markers) in Fraction A, we note unique expression profiles during postnatal development (Fig. 4A). The expression levels of *Gfra1* and *Etv5* remain stable through the three developmental stages (Fig. 4B), with *Gfra1* being constantly at high levels while *Etv5* at medium levels. *Id4* exhibits gradual reduction in its expression levels over time whereas *Nanos2* shows an opposite pattern. These results suggest the heterogeneity in cell composition and a gradual change in the gene expression patterns over time in Fraction A.

### A high-resolution cell fractionation reveals the complex linkage between immunophenotypes and biological functions of the regenerative and SSC culture-initiating cells

**Figure 5:**
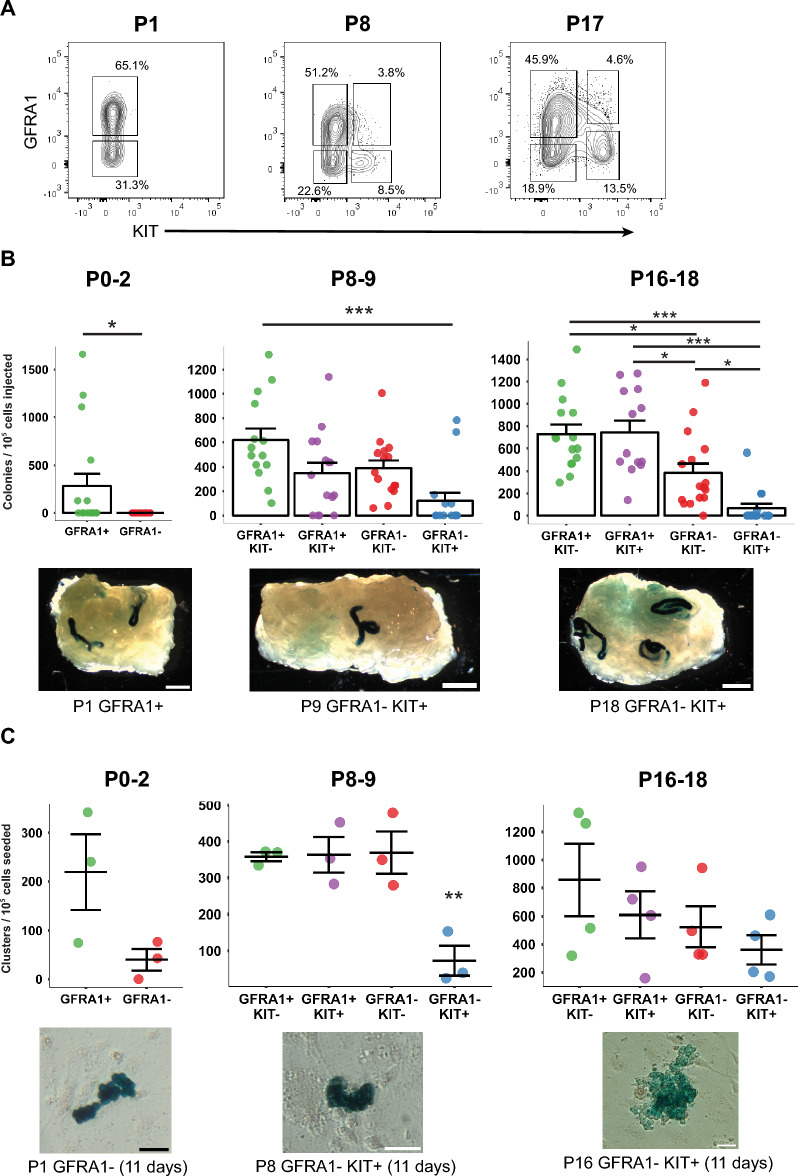
Further fractionation of Fraction A and biological functions of each subfraction. (**A**) Fraction A (i.e., THY1 + ITGA6 +) can be further separated based on GFRA1 and KIT expression. Shown are flow cytometric plots depicting GFRA1 and KIT expression patterns of Fraction A at P1, P9, and P17. GFRA1 and KIT subfraction gates were guided by the GFRA1 and KIT “Fluorescence Minus One” controls. Size (%) of each quadrant is also indicated. (**B**) Regeneration capacity of GFRA1/KIT subfractions as measured by spermatogonial transplantation. Each point represents data from one recipient testis. Significant differences are indicated by asterisks (*p < 0.05; ***p < 0.001). Photographs show images of donor-derived spermatogenic colonies found in recipient testes. Colonies are visualized using X-gal staining. Scale bars: 1 mm. (C) In vitro cluster forming activity of GFRA1/KIT subfractions. Cluster numbers were visually measured after X-gal staining. Significant differences are indicated with asterisks (**p < 0.01). Images of clusters visualized using X-gal staining are shown. Scale bars: 50 µm.

Given the heterogeneity of germ cells in Fraction A (i.e., THY1 + ITGA6 +), we next attempted to further purify regenerative and culture-initiating stem cells in this cell fraction. To this end, we divided Fraction A using GFRA1 and KIT as markers. As shown in Fig. 5A, we detected two cell populations at P0-2: GFRA1 + and GFRA1– cells, with KIT + cells being negligible. At P8-9 and P16-18, four cell fractions constituted Fraction A: GFRA1 + KIT–, GFRA1 + KIT + , GFRA1–KIT–, and GFRA1– KIT + . To our knowledge, this is the first time that mouse pup cells were fractionated using FACS based on the expression of THY1, ITGA6, GFRA1, and KIT together.

Following FACSorting, all cell fractions were individually analyzed for their function to regenerate spermatogenesis upon transplantation and to establish SSC clusters in vitro. As shown in Fig. 5, results of these analyses were similar for both biological functions. At P0-2, only GFRA1 + cells exhibited regenerative capacities (Fig. 5B). In vitro, GFRA1 + cells clearly showed culture-initiating ability at P0-2, although we did not detect a statistical difference when compared to GFRA1-cells (Fig. 5C). At P8-9 and P16-18, GFRA1 + KIT-cells clearly showed the ability to regenerate spermatogenesis and to form clusters while GFRA1-KIT + cells had the lowest level of ability. As such, we observed a trend that throughout the process of postnatal development, GFRA1 + subfractions exhibited greater levels of regenerative and culture-initiating activity than GFRA– subfractions (Fig. 5). Likewise, KIT– subfractions tended to show functional capacities more strongly than KIT + subfractions throughout postnatal development. These trends become more evident when we consider the number of cells included in each subfraction of Fraction A (Supplementary Figure S5). For instance, since GFRA1 + KIT– cells represent 51.2% of Fraction A at P8 (Fig. 5A) and show the highest frequency of regenerative SSCs at this age (Fig. 5B), 77% of the regenerative potential of the Fraction A cells are attributed to GFRA1 + KIT– cells (Supplementary Figure S5).

Significantly, however, all fractions showed a detectable level of SSC functions, except for regenerative capacity in GFRA1-cells at P0-2. For example, although GFRA1-KIT + cells at P8-9 demonstrated the lowest level of regenerative ability, we detected 122.3 ± 65.7 colonies per 10^5^ cells transplanted. This SSC frequency is far greater than that found in unsorted testis cells at neonatal to adult stages; 47-fold higher enrichment compared to unsorted cells at adult age while threefold compared to unsorted cells at P6-8 (Supplementary Table S1). Likewise, the cluster-initiating ability of GFRA1-KIT + cells at P16-18 was roughly a half of the activity that GFRA1 + KIT-cells showed (Fig. 5C). Thus, the Fraction A cells that lost GFRA1 expression and acquired KIT expression have not entirely lost SSC activity, even though this pattern of GFRA1/KIT expression was previously considered to be the indication of the exit from the SSC state^43^. In this context, our data show that despite the high-resolution fractionation, none of the subfractions of Fraction A, at any stage of postnatal development, exhibited a prominently greater magnitude of functional capacity to regenerate spermatogenesis or establish SSC culture than parental Fraction A; i.e., no further SSC enrichment was attained (Figs. 5B and C vs. 2B and 3A). These results emphasize the remarkable heterogeneity in the immunophenotype and function of SSCs.

## Discussion

Although the mouse has been the essential model to understand biology of SSCs and explore technical advancement for SSC applications, functional analyses of these cells over the course of postnatal development has not been documented in detail. Since SSCs are expected to become a critical resource to safeguard the fertility of young boys who undergo sterilizing therapies^2,9^ or to produce, maintain, and propagate favored traits in livestock species^14–16^, we here determined the progress of immunophenotypes of SSCs during development after birth in mice as a model. We identified the cell fractions at different developmental stages that carry the significant capacity to regenerate spermatogenesis upon transplantation and to initiate SSC cultures by which regenerative stem cells are propagated exponentially. A broad spectrum of information presented in this study provides a significant knowledge foundation for future studies and practical applications of SSCs.

This study is unique in two perspectives of experimental approaches. First, although past studies used a genetic marker (e.g., eGFP) for fractionation of immature testis cells in mice^30,31,44^, we relied solely on cell-surface proteins. An approach based on genetic labelling allows for harvesting live cells but does not support the cell fractionation that is ethical or practical in humans and other mammalian species. Using a non-genetic approach in this study, we successfully isolated live and functional SSCs at a significantly high level of enrichment (Figs. 2B and 3A and Supplementary Table S2). Second, our target cells were derived from intact testes without a pretreatment in vivo, such as induced cryptorchidism, which enriches SSCs within testes^22,23^. Artificial in-vivo treatment/pre-enrichment of SSCs is not a feasible option in clinical settings or with livestock species. Our study thus demonstrates that high levels of enrichment for SSCs and culture-initiating cells can be attained effectively from intact testis cells.

Three particularly important findings can be identified in this study. First, the cell fraction that exhibited the highest level of regenerative capacity was the cell fraction that led to the most efficient and effective establishment of long-term SSC propagation culture (Figs. 2B, 3A, and 3B). We previously reported that the number of spermatogonial clusters observed during long-term culture corresponds to that of regenerative stem cells measured by spermatogonial transplantation^36^. While the results of the current study support this observation, it is now well established that the “cell population of origin” of regenerative stem cells is the same as that of culture-initiating cells. Importantly, this indicates that the detection of culture-initiating ability can be used as a surrogate assay to identify regenerative cells. Assuming that this concept applies to humans and other mammalian species, regenerative SSCs or cell fractions that are significantly enriched for such stem cells can be identified by culture even when spermatogonial transplantation is neither a practical nor an ethical option. In fact, such an in-vitro approach has been a common practice in the research of other stem cell types. As a transplantation assay is impossible for stem cells of the central nervous system, neural stem cells are detected by the formation of neurospheres and their maintenance in vitro^45^. Likewise, many types of tumor-initiating cells are identified using tumorsphere assays, an in-vitro technique that has been widely used for drug discovery for the development of anti-cancer therapies^46^.

Second, our data showed that although the frequency of culture-initiating cells changed during the course of postnatal development and gradually increased over time (Fig. 3A), once established, spermatogonial clusters expanded at a similar rate during culture, regardless of the age of origin (Fig. 3B). It is therefore suggested that even if only a limited number of spermatogonial clusters may be derived in vitro at a young age or from a small testis fragment, these established clusters will likely propagate in a manner independent of the age of origin, thereby effectively amplifying regenerative stem cells in vitro.

Third, our study reveals the immunophenotypic heterogeneity of regenerative and culture-initiating stem cells in prepubertal testes (Fig. 5). While these stem cells were enriched in Fraction A (Figs. 2B and 3A), we were able to further separate this cell population into multiple subfractions using GFRA1 and KIT as fractionation parameters (Fig. 5A). When functionally characterized, however, nearly all subfractions possessed regenerative and culture-initiating activities, even though the magnitude of the activities varied across subfractions (Figs. 5B and C). As GFRA1 and KIT are considered to be consensus markers of SSC self-renewal and commitment, respectively^22,47–49^, there was a trend that cells that express GFRA1 showed a greater functional capability than GFRA1– cells, while those that express KIT had less ability than KIT-cells. Nonetheless, we detected regenerative and culture-initiating activities in GFRA- and/or KIT-cells. We speculate that once a target cell population is enriched to a significant level, a consensus SSC marker (be it positive or negative) loses its effectiveness to distinguish functional capabilities of different cell populations. In other words, as long as THY1 and ITGA6 are expressed and when SSC enrichment is achieved at a high level (as in Fraction A), the immunophenotypic heterogeneity obscures the functional disparity detectable. Such an event could arise from technical limitations of flow cytometry, where an extensive cell fractionation could lead to a degree of target cell loss. In addition, SSC fate commitment may proceed gradually, and we do not yet have sufficient knowledge to distinguish different steps of commitment. Possibly, the most adequate markers to monitor SSC fate progression and to purify SSCs are yet to be determined even in mice. In this regard, the process of research development of mouse hematopoietic stem cells (HSCs) is suggestive. So-called KSL cells (Kit + Sca1 + Lineage-cells) had long been accepted as long-term HSCs, but the discovery of SLAM family molecules as novel mouse HSC markers led to an extremely high level of purification of long-term HSCs^50–53^. SSC research may follow this precedent towards purifying these stem cells in the future.

In this context, it was to our surprise that GFRA-KIT-cells showed significant levels of regenerative and culture-initiating abilities (Fig. 5B and C). This result cannot be explained simply by contamination of other cell fractions, since GFRA-KIT-cells clearly showed SSC activity higher than GFRA1–KIT + cells. It is noted that Garbuzov et al.^55^ previously reported that GFRA1– cells have SSC capacity, and that the number of GFRA1– SSCs is similar to that of GFRA1 + SSCs in adult mice. It may be possible that these cells could be immediate progenitors of SSCs that have not completed their commitment and have retained a level of regenerative capacity^37^. It has been reported that NEUROG3-positive cells apparently represent ~ 12% of regenerative cells^54^. We note that the expression level of *Neurog3* transcripts is relatively high in Fraction A at P8-9 and P16-18 (Fig. 4A). More detailed analyses are necessary in the future to address this issue and to determine the biological identity of GFRA-KIT-cells.

Finally, we note that we focused only on germ cells in this study. As shown in Fig. 1C, however, there were multiple cell fractions that did not carry TRA98 + germ cells. Consequently, these fractions showed neither regenerative nor culture-initiating functions (Figs. 2B and 3A). A majority of these non-germ cells did not express THY1 (Fig. 1), further supporting that THY1 is an effective mouse SSC marker. Yet, the identity and biological property of TRA98-cells remain unknown. Analyses based on single-cell RNA sequencing should reveal the cell types of TRA98-cells sorted into different fractions at the three stages of postnatal development. Such analyses should also clarify the heterogeneity of germ cells in Fractions A and C and may further reveal the SSC fate commitment process within and across the cell fractions over time, which should help us to better understand the potential mechanisms of SSC fate control at the transcript level. Such an effort is currently underway in our laboratory, and the data generated will complement the results presented in this study about functional characterization of regenerative/culture-initiating stem cells.

## Methods

### Spermatogonial transplantation

ROSA26 mice (B6;129S-Gt(ROSA)26Sor/J) were used in all experiments, which express β-galactosidase ubiquitously in all tissue including testis^55^. For spermatogonial transplantation, 129/SvEv × C57BL/6 F1 hybrid mice were used as recipients. Four to eight weeks prior to transplantation, recipient mice were intraperitoneally injected at 4–5 weeks of age with busulfan (50 mg/kg of body weight) to abrogate endogenous spermatogenesis. For transplantation, a single cell suspension of donor cells was prepared as in ref. 20. A microinjection needle filled with the donor cell suspension was inserted into the efferent ducts, and donor cells were injected into the lumen of the seminiferous tubules through the rete testis^56,57^. For transplantation assays following FACSorting, six to 12 donor testes were used for each experiment. Testes of recipient mice were recovered 2–3 months after transplantation and stained with X-gal, or 5-bromo-4-chloro-3-indolyl β-D-galactoside (BioBasic) to visualize colonies of donor-derived spermatogenesis^20^. All surgical procedures were done aseptically under isoflurane anesthesia. Following the guidance from the Institutional Animal Care and Use Committee, mice were euthanized using isoflurane and CO2 followed by cervical dislocation.

### Cell culture and the cluster forming assay (CFA)

SSC culture was initiated and maintained as described previously^33,36^: a serum-free medium composed of Minimum Essential Medium α (Invitrogen) with 0.2% BSA (Sigma-Aldrich), 5 µg/ml insulin (Sigma-Aldrich), 10 µg/ml iron-saturated transferrin (Sigma-Aldrich), 3 × 10^–8^ M sodium selenite (Sigma-Aldrich), 50 µM 2-mercaptoethanol (Sigma-Aldrich), 10 mM HEPES (Sigma-Aldrich), 60 µM putrescine (Sigma-Aldrich), 2 mM glutamine (Invitrogen), 50 units/ml penicillin and streptomycin (Invitrogen), and 7.6 µ eq/L free fatty acids (31 mM palmitic acid (Sigma-Aldrich), 2.8 mM palmitoleic acid (Sigma-Aldrich), 11.6 mM stearic acid (Sigma-Aldrich), 13.4 mM oleic acid (Sigma-Aldrich), 35.6 mM linoleic acid (Sigma-Aldrich), 5.6 mM linolenic acid (Sigma-Aldrich)^33^. The following growth factors were added to culture medium, as in Ref. 19: recombinant human GDNF (R & D Systems), recombinant rat GFRA1 (R & D Systems), and recombinant human FGF2 (ThermoFisher). For the initial one or two passages (i.e., initiation stage), 40 ng/ml GDNF, 300 ng/ml GFRA1, and 1 ng/ml FGF2 were added to culture medium. In subsequent maintenance passages, 20 ng/ml GDNF, 75 ng/ml GFRA1, and 1 ng/ml FGF2 were used. Culture medium was replaced with fresh medium and growth factors every 3–4 days, and cells were subcultured following treatment with 0.25% trypsin–EDTA (Invitrogen) for ~ 3 min. SSCs were cultured on a feeder layer of STO (SIM mouse embryo-derived thioguanine and ouabain resistant) fibroblasts^58^ which were mitotically inactivated with mitomycin C (Sigma-Aldrich) treatment (10 μg/ml for 3–3.5 h). STO feeders were seeded in culture plates at 5 × 10^4^ cells/cm^2^ at least one day before germ cell seeding. With the presence of the growth factors and STO feeder cells, undifferentiated spermatogonia proliferate and form three-dimensional cell aggregates or islands within 6 days, which we call “clusters”. All cultures were maintained in 5% CO2 at 37˚C.

The cluster forming assay (CFA)^36,37^ was performed for 6–7 days or 9–11 days depending on the type of experiment and without subculturing. All CFAs were conducted using 48-well plates. In order to count the number of clusters, we defined a cluster as a spermatogonial aggregate formed with at least six cells^36^. Chain-like structures of spermatogonia were excluded as an indication of differentiation^59^. To visualize clusters, wells were fixed with 0.5% glutaraldehyde for at least 5 min, followed by staining with X-gal. Clusters were quantified visually under a light microscope. If clusters were too numerous to manually quantify, the entire well was imaged first, followed by manual quantification.

### Flow cytometry and fluorescence activated cell sorting (FACS)

After recovering mouse testes, tunica was removed and seminiferous tubules were lightly loosened with forceps. The tubules were digested in Hanks’ Balanced Salt Solution with 1 mg/ml Collagenase I (Sigma-Aldrich) and 1 mg/ml Collagenase IV (Sigma-Aldrich) for 10–12 min at 37°C. PBS was added to the collagenase digestion solution to terminate the collagenase reaction. Tubules were sedimented with unit gravity for 10 min for P8-9 or P16-18 pup testes (see later for P0-2). Supernatant was removed and fresh PBS was added to sediment tubules again for 10 min. This process washed off collagenase and a large portion of interstitial cells. For P0-2 pups, testes treated with collagenase were centrifuged at 500 × *g* for 5 min, since seminiferous tubules at this developmental stage are so light that unit gravity sedimentation was impractical. Supernatant was removed after centrifugation. Washing was done by adding PBS and centrifuging again at the same settings. To digest tubules to a single-cell suspension, enzyme-free cell dissociation buffer (Sigma-Aldrich) with 1 mg/ml DNase I (Sigma-Aldrich) was added and incubated for at least 2 min in a 37°C water bath. The digestion was terminated by adding PBS. The resulting cell solution was passed through a 40-μm cell strainer to remove undigested tissue debris.

For flow cytometry, cells were first stained with a viability dye (Biogems Viability Dye 506, Biogems International) in PBS before surface antibody incubation. For incubation in primary antibodies, cells were resuspended in PBS with 1% BSA (Sigma-Aldrich). Up to 2 × 10^6^ cells were resuspended in 100 μl PBS with 1% BSA and incubated with antibodies for 25–35 min on ice with a gentle agitation. One ml of PBS was then added to wash cells, followed by centrifugation at 500 × *g* for 5 min. Finally, cells were resuspended in PBS with 1% BSA at 200–300 μl per 10^6^ cells. Antibodies used for flow cytometry and FACS are summarized in Table 1. The GFRA1 antibody was conjugated to a fluorochrome in house using the Lightning-Link APC conjugation kit (Abcam) with overnight conjugation reaction as recommended by the manufacturer.

**Table 1:** Settings of flow cytometers.

BD LSRFortessa/LSRFortessa X-20/FACSAria fusion								
Laser line	405 nm	405 nm	405 nm	488 nm	488 nm	561 nm	640 nm	640 nm
Emission filter	710/50	525/50	450/50	530/30	530/30	582/15	780/60	670/14
Fluorochrome	BV711	Biogems 506	BV421	FITC	Alexa Fluor 488	PE	APC-Cy7	APC
Antigen	MHC-I	Viability	KIT	CD45	CD74	THY1	ITGA6	GFRA1

Flow cytometric analyses were done using the BD (Becton, Dickinson and Company) LSRFortessa or LSRFortessa X-20. For FACS, cells were sorted on the BD FACSAria Fusion. Table 2 details the setup of flow cytometric instruments. Data were analyzed with FlowJo™ v10.7 or v10.8 Software (BD Life Sciences).

**Table 2:** List of antibodies used.

Antigen	Fluorochrome	Vendor	Catalog #	Stock conc	Working conc	Application
THY1	PE	Biolegend	105,308	0.2 mg/mL	1:200	Flow
ITGA6	APC-CY7	Biolegend	313,628	0.1 mg/ml	1:200	Flow
GFRA1	APC	R&D	MAB560	1 mg/mL	1:200	Flow
KIT	BV421	Biolegend	105,827	0.05 mg/mL	1:333	Flow
MHC-I (H-2K^b^/H-2D^b^)	BV711	BD	745,433	0.2 mg/mL	1:333	Flow
CD45	FITC	Invitrogen	11–0452-82	0.5 mg/mL	1:333	Flow
CD74	Alexa Fluor 488	Biolegend	151,006	0.5 mg/mL	1:100	Flow
TRA98	N/A	B-Bridge	73–003	1 mg/mL	1:200	Intra. Flow

Flow: flow cytometry; Intra. Flow: intracellular flow cytometry.

### Intracellular flow cytometry

First, surface staining was performed as above. Cells were then fixed in ice-cold 4% paraformaldehyde, followed by a 15-min incubation on ice with gentle agitation^60^. Cells were washed twice with PBS with centrifugation at 500 × *g* for 5 min each and permeabilized using ice-cold 0.1% Triton X-100 for 5 min on ice. Cells were then washed using PBS with centrifugation at 500 × *g* for 5 min and incubated in blocking buffer (1% BSA in PBS) for 5 min on ice. After the blocking process, cells were aliquoted for staining with a given antibody and centrifuged at 500 × *g* for 5 min. Primary antibodies (100 μl total volume / tube) were then added to the cells resuspended in PBS with 1% BSA. The sample tubes were incubated for 20 min. After a 20-min incubation on ice with agitation, 1 ml PBS was added to wash each sample followed by centrifugation at 500 × *g* for 5 min. Then, cells were resuspended in 1% BSA-PBS with secondary antibodies (1:500 Donkey anti-Rat IgG (H + L) Highly Cross-Adsorbed Secondary Antibody, Alexa Fluor 488, Invitrogen) and incubated for 10 min on ice with agitation. Cells were washed with 1 ml PBS and centrifugation as above. Cells were resuspended in 200–300 μl of 1% BSA-PBS and analyzed on the BD LSRFortessa or LSRFortessa X-20.

Intracellular staining for TRA98 was performed as described above, and cells were analyzed with flow cytometry. To integrate TRA98 staining onto THY1-ITGA6 profiles (Fig. 1C), TRA98-positive cells were backgated onto the THY1/ITGA6 profiles in FlowJo software.

### Quantitative real-time PCR (RT-qPCR)

The PicoPure RNA isolation kit (ThermoFisher) was used to extract and isolate total RNA from FACS-sorted cells. Contaminating DNA was removed by using the RNase-Free DNase set (Qiagen). Isolated RNA was evaluated for quality and RNA concentration using the NanoDrop 2000 spectrophotometer (ThermoFisher) and 25 ng of starting RNA was used for cDNA synthesis using Superscript III Reverse Transcriptase (Invitrogen), Anchored Oligo dT Primers (ThermoFisher), and RNaseOUT (ThermoFisher). Quantitative PCR was performed using the BlasTaq 2X qPCR MasterMix (Applied Biological Materials (abm) Inc.) on the Roche LightCycler 96, and associated software was used for data analysis. Cycling settings were 3 min for preincubation at 95°C followed by 40 cycles of 95°C for 15s for denaturation / 55°C for 30s for annealing / 60°C for 30s for elongation. Primers are detailed in Table 3. Beta-actin (*Actb*) was used as a reference gene. Statistical differences are indicated by distinct alphabets (p < 0.05) in Fig. 4B and Fig. S4.

**Table 3:** List of primers used for RT-qPCR.

Gene	Forward primer	Reverse primer	References
*Gfra1*	GTGGCAATGACCTGGAAGAT	ATTGCCAAAGGCTTGAATTG	49
*Id4*	CTACCATCCCGCCCAACAAG	CTCAGCAAAGCAGGGTGAGT	Original
*Etv5*	CATCCTACATGAGAGGCGGG	TCCTGCTTGACTTTGCCTTCC	Original
*Nanos2*	TCCCATCCTGAGGCACTATGT	ACTGCTGTTGAGTGGACAATAC	62
*Zbtb16*	CGAGCTTCCGGACAACGA	TTGGCACCCGCTGAATG	63
*Nanos3*	TGTAAGGCTGGATCCCAAAC	CTGATAGATGGCACGGGACT	Original
*Neurog3*	GCTATCCACTGCTGCTTGA	CCGGGAAAAGGTTGTTGTGT	64
*Sohlh2*	GGATTAAAGGCCCCGTTGTC	ATCGCTCTTCCTCCCCTTGA	65
*Sohlh1*	AGCGGGCCAATGAGGATTAC	CTGCGTTCTCTCTCGCTGAC	66
*Kit*	TGGGAGTTTCCCAGAAACAG	AAATGGGCACTTGGTTTGAG	67
*Stra8*	CTCTCCCACTCCTCCTCCA	GAGGTCCATGGTCTGCTTGTA	68
*Wt1*	GAGAGCCAGCCTACCATCC	GGGTCCTCGTGTTTGAAGGAA	Original
*Actb*	CCCTAAGGCCAACCGTGAAA	AGCCTGGATGGCTACGTACA	68

### Statistical analyses

Both data in figures and the text were presented as mean ± standard error of the mean (SEM). To compare the means of groups, one-way analysis of variance (ANOVA) or Welch’s t-test (if only two groups) was conducted. For post hoc pairwise comparisons, Tukey’s HSD was used. Statistical analyses and generation of figures were done in the R (4.2.1) software environment.

### Ethics statement

All procedures of care and experimental handling of mice as described in this study followed the guideline determined by the Canadian Council on Animal Care and were approved by the Institutional Animal Care and Use Committee of the Research Institute of the McGill University Health Centre.

## Data availability statement

Any data not available within the article and/or supplementary materials can be requested to the corresponding author (MN).

### Supplementary Information

Supplementary Information.
