# Supplementary material for: Postnatal development of mouse spermatogonial stem cells as determined by immunophenotype, regenerative capacity, and long-term culture-initiating ability: a model for practical applications
Source: Sci Rep. 2024 Jan 27;14:2299. doi: 10.1038/s41598-024-52824-8 (PMC10821885; doi:10.1038/s41598-024-52824-8)
Supplement: Supplementary file 1 — Supplementary Information. [file 41598_2024_52824_MOESM1_ESM.pdf]

Table S1: Weight, cell recovery, and number of colonies per testis increases with age in mouse. T: testis, Col: colonies. Three replicates of transplantation assays were performed for each age group. The values of "Cell Recovery# / T" are the average of cell numbers counted after digesting the testes into a single cell suspension divided by the number of donor testes used. For transplantation, 7 – 8 μl of cell suspension were injected into each recipient testis. The cell concentrations injected were 100x 10^6 cells/ml (adult, 2 donor testes/experiment), 20 x 10^6 cells/ml (P16-18, 2 to 4 donor testes/experiment), 10 x 10^6 cells/ml (P6-8, 2 to 6 donor testes/experiment)) and 38.4 to 50 x 10^6 cells/ml (P0-2, 4 to 14 donor testes/experiment).

Age	Col#/ 10^5 cells	Col#/ Donor T	% of Adult	Average T weight	Col#/ mg testis	Cell Recovery#/ T (x 10^6)
**Adult**	2.6 ± 0.2	736.5	100%	101.4 mg	7.3 col/mg	27.7
**P16-18**	15.4 ± 1.9	392.0	53.2%	13.6 mg	28.8 col/mg	2.7
**P6-8**	39.0 ± 2.5	277.8	37.7%	1.7 mg	168.3 col/mg	0.8
**P0-2**	6.5 ± 0.7	31.9	4.3%	0.6 mg	49.8 col/mg	0.5

Table S2: SSC enrichment in Fraction A. Fold increase was calculated by comparing to data of unsorted cells in Table S1. Data for P8-9 Fraction A was compared to P6-8 data from Table S1.

Age & Fraction	Colonies per 10^5 transplanted	Fold increase	SSC frequency (Fraction A)	SSC frequency (unsorted)
**P0-2 Frac. A**	238.3	36.7	1 SSC in 50 cells	1 SSC in 1846 cells
**P8-9 Frac. A**	669.2	17.2	1 SSC in 18 cells	1 SSC in 308 cells
**P16-18 Frac. A**	618.1	40.1	1 SSC in 19 cells	1 SSC in 779 cells

Table S3: Previous reports of high levels of SSC enrichment from mouse pups.

Age	Method	Cell population	Colonies per 10^5 cells transplanted	Reference
**P8**	Transgene	ID4-EGFP^{Bright}	~180	31
**P6**	Transgene + FACS	ID4-EGFP^{+} TSPAN8^{High}	~240	30
**P6**	MACS	THY1+	228	34
**P4.5-5.5**	FACS	THY1+ITGAV−	342	32
**P0-2**	FACS	THY1+ITGAV−	17	32

Table S4: List of long-term culture lines generated by sorting Fraction A. "Doubling time" is the number of days calculated for the number of clusters to double. "Total fold expansion" refers to growth in the number of clusters, compared to the number of clusters at P0 (after 6-7 days in vitro).

Pup Age	Length of culture period	Doubling time (days)	Total fold expansion
**P1**	91 days (10 passages)	3.70	6.82×10^6
**P1**	85 days (9 passages)	3.86	1.81×10^6
**P0**	81 days (10 passages)	3.12	1.70×10^7
**P9**	72 days (9 passages)	3.29	8.41×10^5
**P9**	75 days (9 passages)	2.94	1.08×10^7
**P17**	87 days (11 passages)	3.98	1.28×10^6
**P16**	78 days (9 passages)	3.49	1.51×10^6

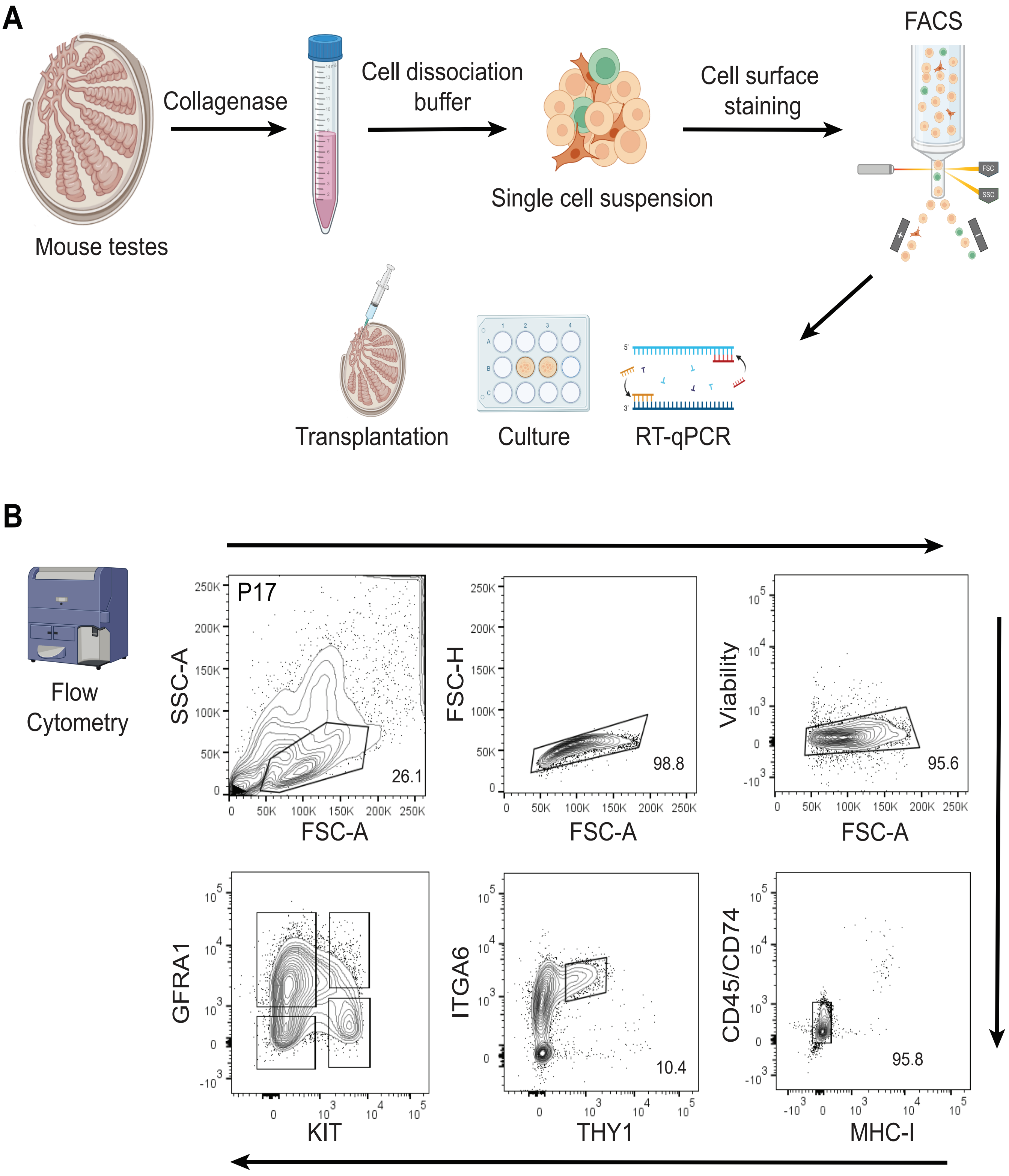

Figure S1: Experimental procedures of this study.
(A) Schematic representation of the study. Illustration created with BioRender.com. **(B)** Steps taken during flow cytometric profiling. The panel shows, from the top left to bottom left, the selection of low side-scatter cells, single cells, and live cells, followed by a negative selection, profiling based on expression of THY1 and ITGA6, and further fractionation according to expression of GFRA1 and KIT.

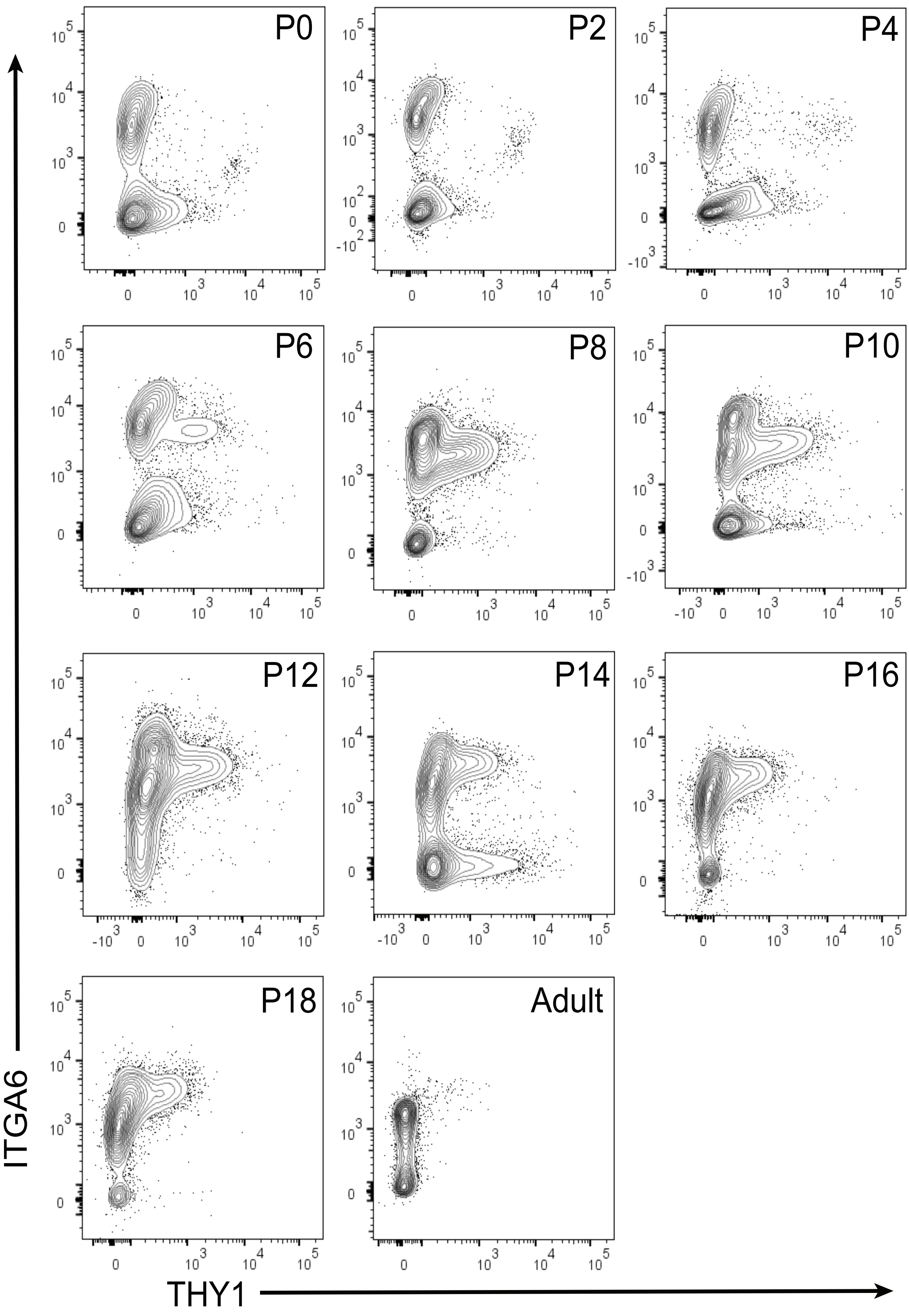

Figure S2: Progression of flow cytometric profiles of mouse testis cells during postnatal development.

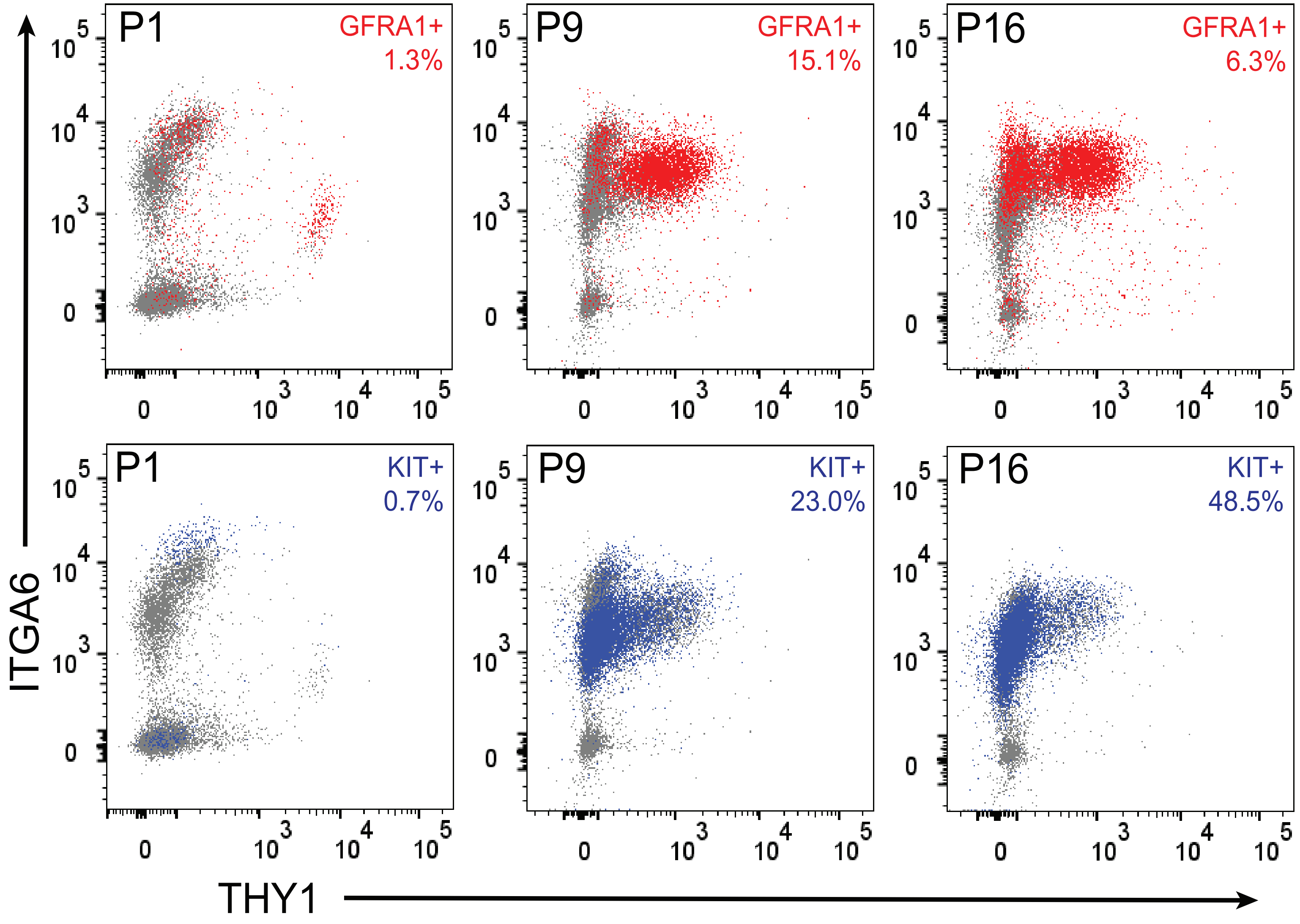

Figure S3: Localization of GFRA1+ and KIT+ cells on flow cytometric profiles.

GFRA1+ cells (top, labeled in red) and KIT+ cells (bottom, in blue) are backgated onto the THY1/ITGA6 flow cytometric profiles using Flowjo software. The proportion of each cell type relative to the entire cells plotted on the profile chart is also shown.

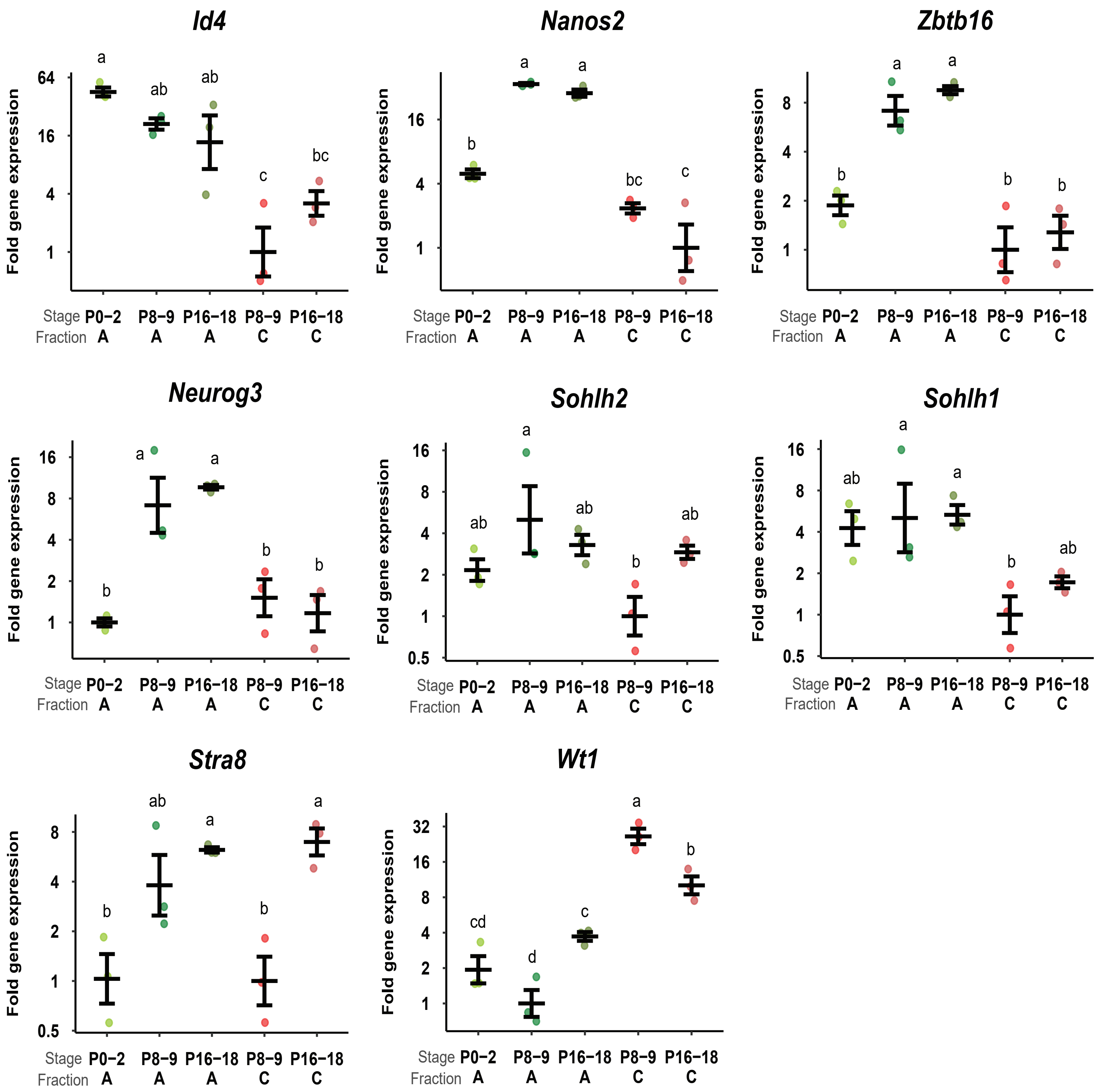

Figure S4: Expression of spermatogonial marker genes.

The results of eight genes are presented, as in Fig. 4B. Statistical differences are indicated by distinct alphabets ($p < 0.05$). For example, there was a significant difference detected between data labeled 'ab' and 'c', but there was no significant difference between data labeled 'ab' and 'bc'.

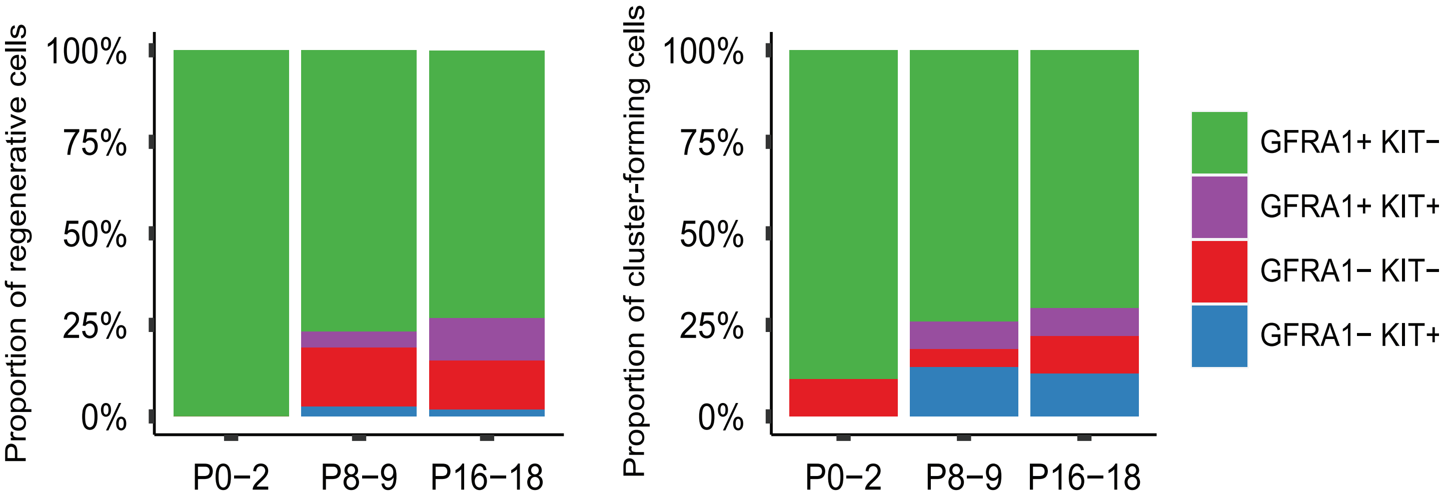

Stage	fraction	proportion
P0-2	GFRA1+ KIT-	100%
P0-2	GFRA1- KIT-	0%
P8-9	GFRA1+ KIT-	77%
P8-9	GFRA1+ KIT+	4%
P8-9	GFRA1- KIT-	16%
P8-9	GFRA1- KIT+	3%
P16-18	GFRA1+ KIT-	73%
P16-18	GFRA1+ KIT+	12%
P16-18	GFRA1- KIT-	13%
P16-18	GFRA1- KIT+	2%

Stage	fraction	proportion
P0-2	GFRA1+ KIT-	90%
P0-2	GFRA1- KIT-	10%
P8-9	GFRA1+ KIT-	74%
P8-9	GFRA1+ KIT+	8%
P8-9	GFRA1- KIT-	5%
P8-9	GFRA1- KIT+	13%
P16-18	GFRA1+ KIT-	70%
P16-18	GFRA1+ KIT+	8%
P16-18	GFRA1- KIT-	10%
P16-18	GFRA1- KIT+	12%

Figure S5: Population size of regenerative cells (left) and culture-initiating cells (right) in each subfraction at three stages of postnatal development.

(Top) The relative population sizes (%) of functional cells in different subfractions (color-coded) in Fraction A are shown. These values are calculated as SSC activity (colony numbers/10^5 cells transplanted, left) or cluster-forming activity (cluster numbers/10^5 cells placed in culture, right) in each subfraction multiplied by the % of cells in each subfraction among all cells in Fraction A.

(Bottom) Tables showing the average values of each subfraction, as presented in the Top figures.
